# Supplementary material for: Acetate attenuates hyperoxaluria-induced kidney injury by inhibiting macrophage infiltration via the miR-493-3p/MIF axis
Source: Commun Biol. 2023 Mar 15;6:270. doi: 10.1038/s42003-023-04649-w (PMC10017675; doi:10.1038/s42003-023-04649-w)
Supplement: Supplementary file 2 — Supplementary Information [file 42003_2023_4649_MOESM2_ESM.pdf]

Supplementary figure 1 – Uncropped blot images

Figure 1, f

IL-6

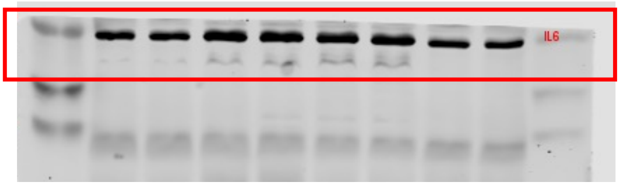

TGF-β

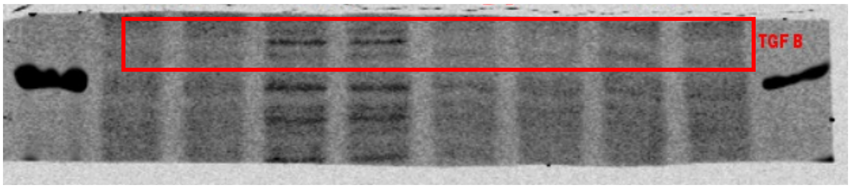

IL-1β

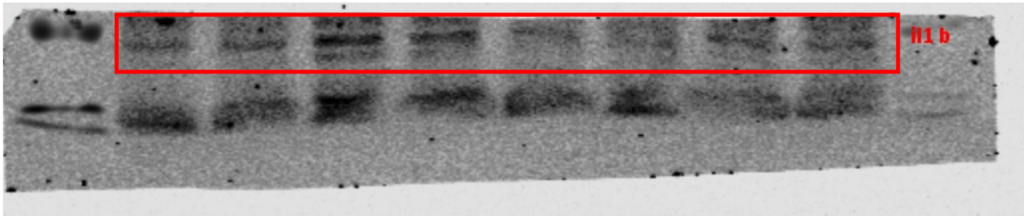

α-SMA

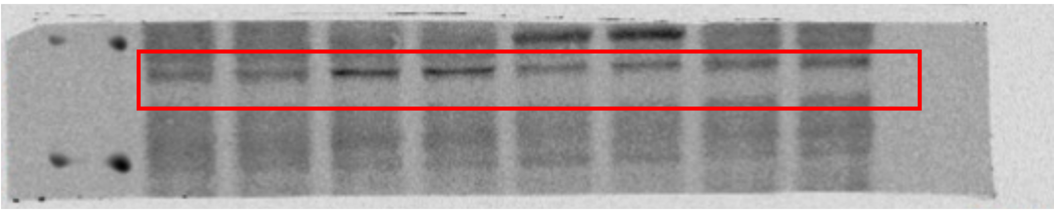

fn1

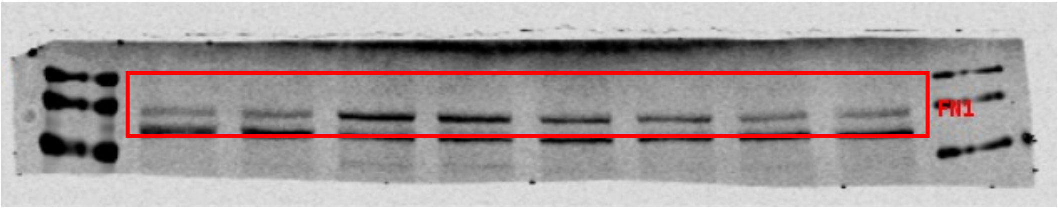

Col3a1

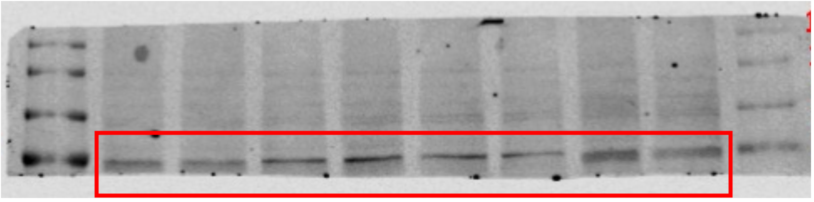

(Continued)

GAPDH

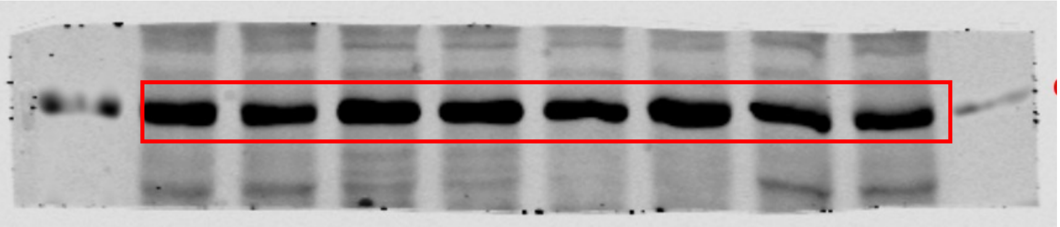

Figure 3, a

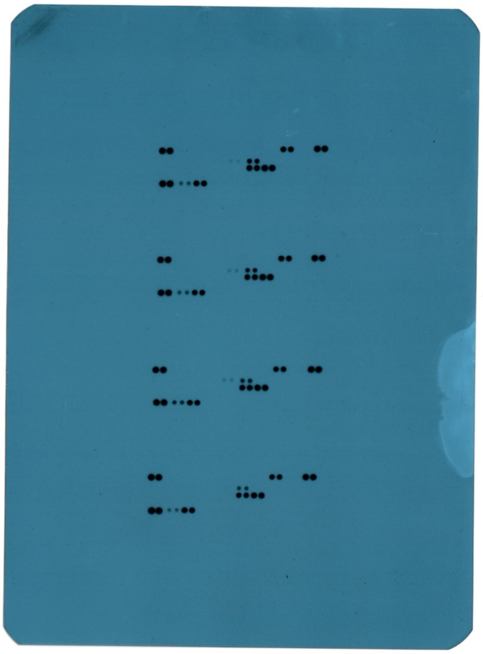

Ac

Ox+Ac

Ox

Ctrl

Figure 4, e

HK-2 MIF

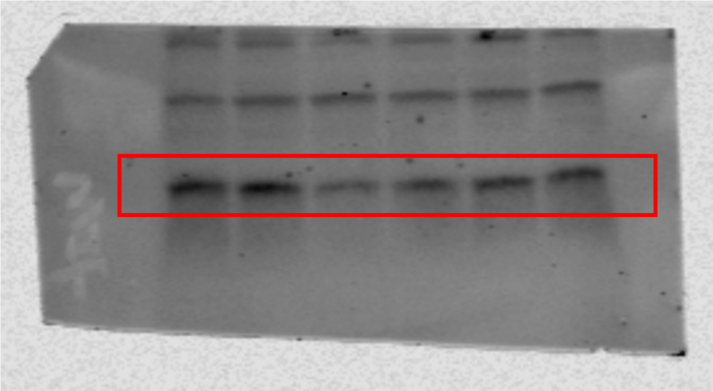

(Continued)

HK-2 GAPDH

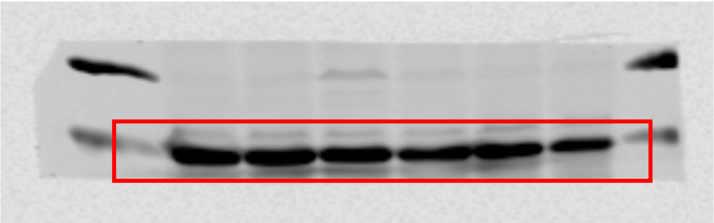

M-1 MIF

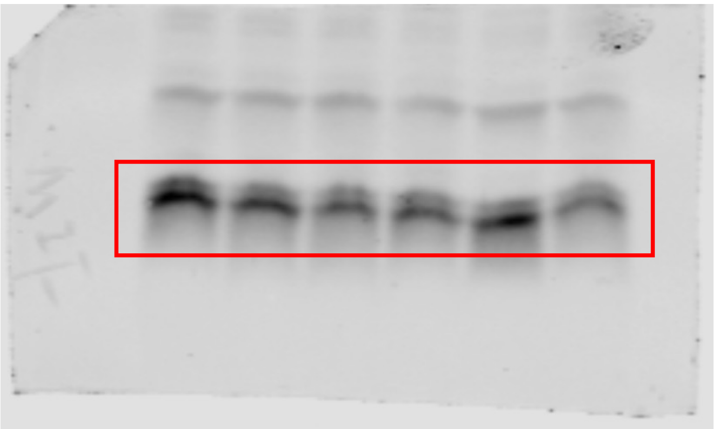

M-1 GAPDH

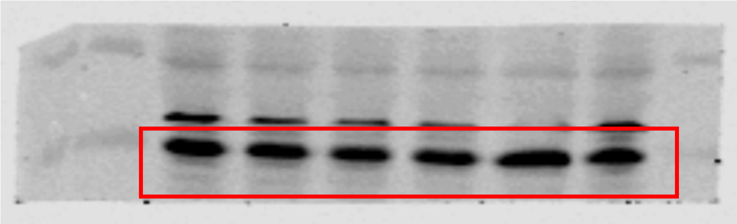

Figure 4, g

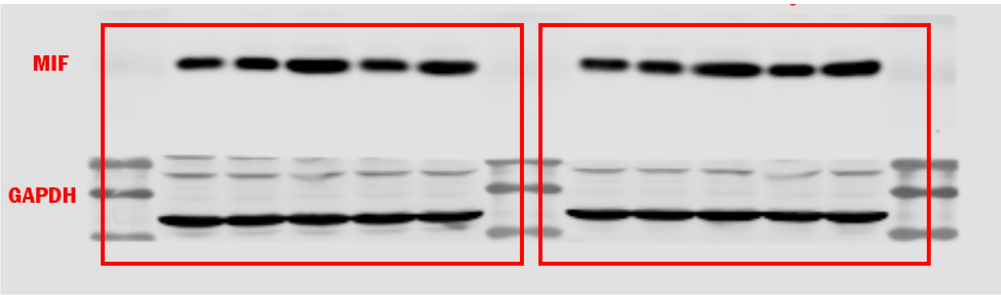

HK-2 M-1

Figure 5, a

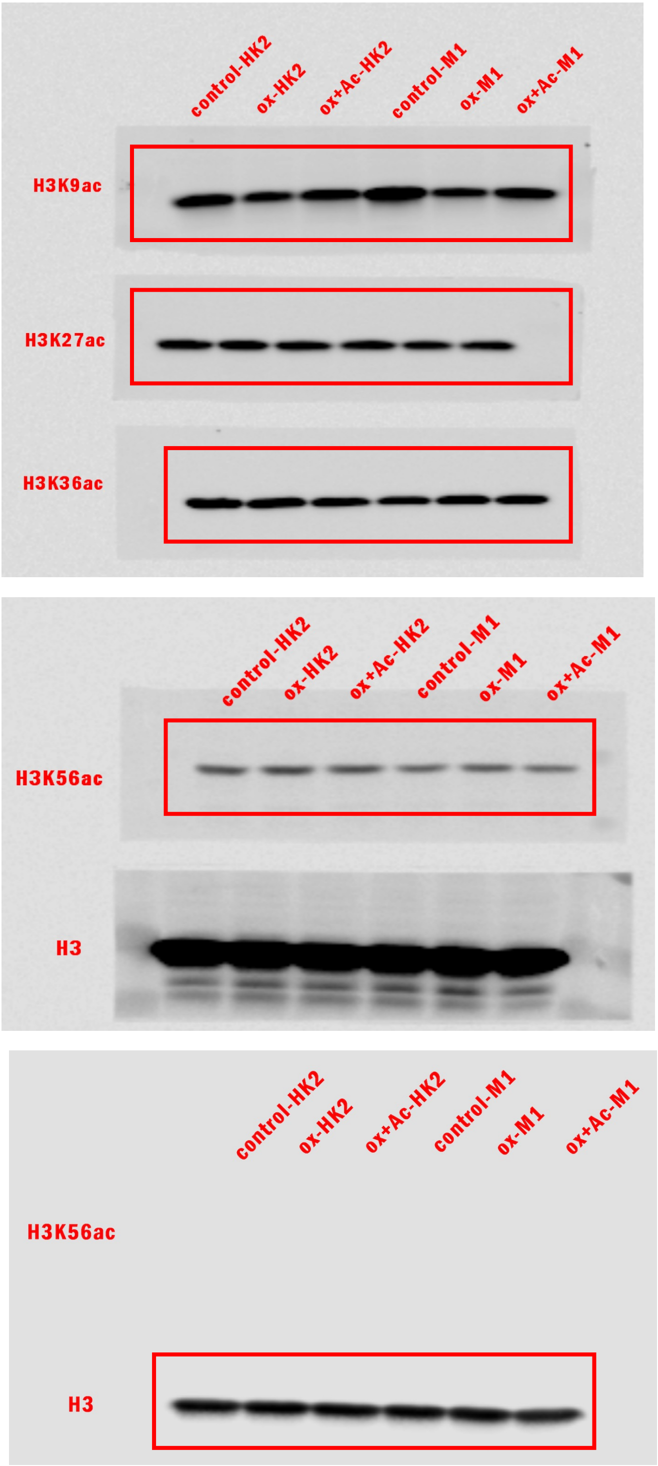
control-HK2
ox-HK2
ox+Ac-HK2
control-M1
ox-M1
ox+Ac-M1
H3K9ac
H3K27ac
H3K36ac
control-HK2
ox-HK2
ox+Ac-HK2
control-M1
ox-M1
ox+Ac-M1
H3K56ac
H3
control-HK2
ox-HK2
ox+Ac-HK2
control-M1
ox-M1
ox+Ac-M1
H3K56ac
H3

Supplementary Table 1 – antibody used in this study

**Vendor**	Antibody	**Host species**	**Catalog number**	**Use**	**Working dilution**
Abcam	Anti-8ohdg	Mouse	ab48508	IHC	1:100
	Anti-αSMA	Mouse	ab7817	IHC/WB	1:100
	Anti-IL-1β	Rabbit	ab254360	IHC/WB	1:100
	Anti-TNFα	Mouse	ab1793	IHC	1:100
	Anti-MIF	Rabbit	ab65869	IHC/WB	1:200/1:1000
	Anti-CD86	Mouse	ab220188	IHC	1:100
	Anti-CD68	Rabbit	ab125212	IHC	1:100
	Anti-H3K9ac	Rabbit	ab4441	ChIP/IHC/WB	1:50/1:100/1:1000
	Anti-H3K27ac	Rabbit	ab4729	ChIP/IHC/WB	1:50/1:100/1:1000
Cell Signaling	Anti-H3	Rabbit	#9715	WB	1:1000
	Anti-H3K56ac	Rabbit	#4243	WB	1:1000
	Anti-H3K36ac	Rabbit	#27683	WB	1:1000
Santa Cruz	Anti-GAPDH	Mouse	sc-47724	WB	1:2000
Proteintech	Anti-COL3A1	Rabbit	22734-1-AP	WB	1:1000
	Anti-TGFβ	Rabbit	21898-1-AP	WB	1:5000
	Anti-FN1	Rabbit	15613-1-AP	WB	1:10000
Abclonal	Anti-IL6	Rabbit	A0286	WB	1:2000

WB, Western blot; IHC, immunohistochemistry; ChIP, chromatin immunoprecipitation.

Supplementary Table 2 - Primers used in this study

Sequences of primers targeting genes for Q-PCR

Species	Gene	Forward primer (5' to 3')	Reverse primer (5' to 3')
Rat	Gapdh	TCTCTGCTCCTCCCTGTTC	ACACCGACCTTCACCATCT
Rat	Fn1	GTAGGCAACACTGTCAACG	AAAGCCAGAGTCAGATAACC
Rat	Tgfα	CCACGCTCTTCTGTCTACTG	GCTACGGGCTTGTCACTC
Rat	αSMA	ACTGCTGCTTCCTCTTCTT	GCTGTTATAGGTGGTTTCG
Rat	Col3a1	CGGAGAATACTGGGTTGA	AGGATTGCCATAGCTGAA
Rat	Il4	TTACGGCAACAAGGAACA	GCACGGAGGTACATCACG
Rat	CD68	TTCAAACAGGACCGACAT	ATTGCTGGAGAAAGAACTATG
Rat	CD44	GAAGGGACTGTTGCTACTGA	AGGAGGGATGCCAAGATG
Rat	CTGF	GCGGCGAGTCCTTCCAAA	CACCCACTCCTCACAGCATTTC
Rat	Il1β	GATGATGACGACCTGCTA	TTGTTGGCTTATGTTCTGT
Rat	Fsp1	TGTAATAGTGTCCACCTTCC	CATTGTCCCTGTTGCTGT
Rat	Tgfβ	TGAACCAAGGAGACGGAATA	CTGTGCAGGTGTTGAGCC
Rat	Col1a1	TGAGCCAGCAGATTGAGAA	GGTTGCAGCCTTGGTTAG
Rat	Il6	CCTTCTTGGGACTGATGT	ACTGGTCTGTTGTGGGTG
Rat	KIM	TGGAGATTCCTGGATGGT	GAGGTGGAGACTCTGGTTGA
Rat	MIF	ATGAACGCAGCCAACGTGG	GGGTGGATAAACACAGAACGGG
Mouse	Gapdh	AAGAAGGTGGTGAAGCAGG	GAAGGTGGAAGAGTGGGAGT
Mouse	Ccl-2	TAAAAACCTGGATCGGAACCAAA	GCATTAGCTTCAGATTTACGGGT
Mouse	Ccl-3	TGTACCATGACACTCTGCAAC	CAACGATGAATTGGCGTGGAA
Mouse	Ccl-4	TTCCTGCTGTTTCTCTTACACCT	CTGTCTGCCTCTTTTGGTCAG
Mouse	Ccl-5	GCTGCTTTGCCTACCTCTCC	TCGAGTGACAAACACGACTGC
Mouse	Il1β	GAAATGCCACCTTTTGACAGTG	TGGATGCTCTCATCAGGACAG

Mouse	Tnfα	CTGAACTTCGGGGTGATCGG	GGCTTGTCACTCGAATTTTGAGA
Mouse	Opn	AGCAAGAAACTCTTCCAAGCAA	GTGAGATTCGTCAGATTCATCCG
Mouse	MIF	GCAAGCCCGCACAGTACAT	GCGTTCATGTCGTAATAGTTGATGTA
Human	Ccl-2	CAGCCAGATGCAATCAATGCC	TGGAATCCTGAACCCACTTCT
Human	Ccl-3	AGTTCTCTGCATCACTTGCTG	CGGCTTCGCTTGGTTAGGAA
Human	Ccl-4	TCGCAACTTTGTGGTAGA	TTCAGTTCCAGGTCATACAC
Human	Ccl-5	TGCTTTGCCTACATTGCC	CCTTGACCTGTGGACGACT
Human	Il-1β	ATGATGGCTTATTACAGTGGCAA	GTCGGAGATTCGTAGCTGGA
Human	Tnfα	GAGGCCAAGCCCTGGTATG	CGGGCCGATTGATCTCAGC
Human	Opn	GAAGTTTCGCAGACCTGACAT	GTATGCACCATTCAACTCCTCG
Human	MIF	TCCGAGAAGTCAGGCACGTAG	TGCACCGCGATGTACTGG
Human	Gapdh	GGAGCGAGATCCCTCCAAAAT	GGCTGTTGTCATACTTCTCATGG

Sequences for primers targeting miR-493-3p promoter region in ChIP-qPCR assay

Gene	Forward primer (5' to 3')	Reverse primer (5' to 3')
miR-493-3p	GATGCTGTCGTGGGGTAGAT	CTTCCACCATCAGCCAACAC
