# Supplementary material for: Acetate attenuates hyperoxaluria-induced kidney injury by inhibiting macrophage infiltration via the miR-493-3p/MIF axis
Source: Commun Biol. 2023 Mar 15;6:270. doi: 10.1038/s42003-023-04649-w (PMC10017675; doi:10.1038/s42003-023-04649-w)
Supplement: Supplementary file 5 — Reporting Summary [file 42003_2023_4649_MOESM5_ESM.pdf]

nature portfolio

Corresponding author(s): Guohua Zeng

Last updated by author(s): Nov 22, 2022

Reporting Summary

Nature Portfolio wishes to improve the reproducibility of the work that we publish. This form provides structure for consistency and transparency in reporting. For further information on Nature Portfolio policies, see our Editorial Policies and the Editorial Policy Checklist.

Statistics

For all statistical analyses, confirm that the following items are present in the figure legend, table legend, main text, or Methods section.

n/a	Confirmed	
☒	☐	The exact sample size (*n*) for each experimental group/condition, given as a discrete number and unit of measurement
☐	☒	A statement on whether measurements were taken from distinct samples or whether the same sample was measured repeatedly
☐	☒	The statistical test(s) used AND whether they are one- or two-sided<br>*Only common tests should be described solely by name; describe more complex techniques in the Methods section.*
☐	☒	A description of all covariates tested
☐	☒	A description of any assumptions or corrections, such as tests of normality and adjustment for multiple comparisons
☒	☐	A full description of the statistical parameters including central tendency (e.g. means) or other basic estimates (e.g. regression coefficient) AND variation (e.g. standard deviation) or associated estimates of uncertainty (e.g. confidence intervals)
☒	☐	For null hypothesis testing, the test statistic (e.g. *F*, *t*, *r*) with confidence intervals, effect sizes, degrees of freedom and *P* value noted<br>*Give P values as exact values whenever suitable.*
☒	☐	For Bayesian analysis, information on the choice of priors and Markov chain Monte Carlo settings
☒	☐	For hierarchical and complex designs, identification of the appropriate level for tests and full reporting of outcomes
☒	☐	Estimates of effect sizes (e.g. Cohen's *d*, Pearson's *r*), indicating how they were calculated

Our web collection on statistics for biologists contains articles on many of the points above.

Software and code

Policy information about availability of computer code

Data collection	Unicel DxC 600 synchronic biochemical detecting system<br>Bio-Rad CFX96 system
Data analysis	Prism 8

For manuscripts utilizing custom algorithms or software that are central to the research but not yet described in published literature, software must be made available to editors and reviewers. We strongly encourage code deposition in a community repository (e.g. GitHub). See the Nature Portfolio guidelines for submitting code & software for further information.

Data

Policy information about availability of data

All manuscripts must include a data availability statement. This statement should provide the following information, where applicable:

- Accession codes, unique identifiers, or web links for publicly available datasets
- A description of any restrictions on data availability
- For clinical datasets or third party data, please ensure that the statement adheres to our policy

Guohua Zeng had full access to all the data in the study and takes responsibility for the data and the accuracy of the data analysis.

Human research participants

Policy information about studies involving human research participants and Sex and Gender in Research.

Reporting on sex and gender	N/A
Population characteristics	N/A
Recruitment	N/A
Ethics oversight	N/A

Note that full information on the approval of the study protocol must also be provided in the manuscript.

Field-specific reporting

Please select the one below that is the best fit for your research. If you are not sure, read the appropriate sections before making your selection.

☒ Life sciences ☐ Behavioural & social sciences ☐ Ecological, evolutionary & environmental sciences

For a reference copy of the document with all sections, see nature.com/documents/nr-reporting-summary-flat.pdf

Life sciences study design

All studies must disclose on these points even when the disclosure is negative.

Sample size	No sample-size calculation was performed. 6 rats per groups were enough as significant results were obtained.
Data exclusions	No data were excluded from the analyses.
Replication	All attempts at replication were successful.
Randomization	This is not relevant to the study.
Blinding	This is not relevant to the study.

Reporting for specific materials, systems and methods

We require information from authors about some types of materials, experimental systems and methods used in many studies. Here, indicate whether each material, system or method listed is relevant to your study. If you are not sure if a list item applies to your research, read the appropriate section before selecting a response.

Materials & experimental systems

n/a	Involved in the study	
☐	☒	Antibodies
☐	☒	Eukaryotic cell lines
☒	☐	Palaeontology and archaeology
☐	☒	Animals and other organisms
☒	☐	Clinical data
☒	☐	Dual use research of concern

Methods

n/a	Involved in the study	
☒	☐	ChIP-seq
☒	☐	Flow cytometry
☒	☐	MRI-based neuroimaging

Antibodies

Antibodies used

Abcam	Anti-8ohdg	ab48508
Abcam	Anti-αSMA	ab7817
Abcam	Anti-IL-1β	ab254360
Abcam	Anti-TNFα	ab1793
Abcam	Anti-MIF	ab65869
Abcam	Anti-CD86	ab220188
Abcam	Anti-CD68	ab125212
Abcam	Anti-H3K9ac	ab4441

Abcam Anti-H3K27ac ab4729
Cell Signaling Anti-H3 #9715
Cell Signaling Anti-H3K56ac #4243
Cell Signaling Anti-H3K36ac #27683
Santa Cruz Anti-GAPDH sc-47724
Proteintech Anti-COL3A1 22734-1-AP
Proteintech Anti-TGFβ 21898-1-AP
Proteintech Anti-FN1 15613-1-AP
Abclonal Anti-IL6 A0286

Validation

Abcam Anti-8ohdg Mouse ab48508 IHC
Abcam Anti-αSMA Mouse ab7817 IHC/WB
Abcam Anti-IL-1β Rabbit ab254360 IHC/WB
Abcam Anti-TNFα Mouse ab1793 IHC
Abcam Anti-MIF Rabbit ab65869 IHC/WB
Abcam Anti-CD86 Mouse ab220188 IHC
Abcam Anti-CD68 Rabbit ab125212 IHC
Abcam Anti-H3K9ac Rabbit ab4441 ChIP/IHC/WB
Abcam Anti-H3K27ac Rabbit ab4729 ChIP/IHC/WB
Cell Signaling Anti-H3 Rabbit #9715 WB
Cell Signaling Anti-H3K56ac Rabbit #4243 WB
Cell Signaling Anti-H3K36ac Rabbit #27683 WB
Santa Cruz Anti-GAPDH Mouse sc-47724 WB
Proteintech Anti-COL3A1 Rabbit 22734-1-AP WB
Proteintech Anti-TGFβ Rabbit 21898-1-AP WB
Proteintech Anti-FN1 Rabbit 15613-1-AP WB
Abclonal Anti-IL6 Rabbit A0286 WB

Eukaryotic cell lines

Policy information about cell lines and Sex and Gender in Research

Cell line source(s)

The human proximal tubular epithelial HK-2 cells, human monocyte THP-1 cells, human embryonic kidney cell line HEK-293T, mouse macrophage RAW264.7 cells, and mouse cortical collecting duct M-1 cells were purchased from the American Type Culture Collection (ATCC) (Rockville, MD).

Authentication

All cell lines used in the study were authenticated by ATCC.

Mycoplasma contamination

All cell lines tested negative for my mycoplasma contamination.

Commonly misidentified lines
(See ICLAC register)

N/A

Animals and other research organisms

Policy information about studies involving animals; ARRIVE guidelines recommended for reporting animal research, and Sex and Gender in Research

Laboratory animals

Male Sprague Dawley rats, aged 6-8 weeks,

Wild animals

The study did not involve wild animals.

Reporting on sex

All the animals in the study were male.

Field-collected samples

the study did not involve samples collected from the field.

Ethics oversight

Ethics committee of the First Affiliated Hospital of Guangzhou Medical University

Note that full information on the approval of the study protocol must also be provided in the manuscript.
